# *P450* Pharmacogenetics in Indigenous North American Populations

**DOI:** 10.3390/jpm8010009

**Published:** 2018-02-01

**Authors:** Lindsay M. Henderson, Katrina G. Claw, Erica L. Woodahl, Renee F. Robinson, Bert B. Boyer, Wylie Burke, Kenneth E. Thummel

**Affiliations:** 1 Departments of Pharmaceutics, University of Washington, Seattle, WA 98195, USA; lmhender@uw.edu (L.M.H.); kclaw@uw.edu (K.G.C.); 2 Department of Biomedical and Pharmaceutical Sciences, University of Montana, Missoula, MT 59812, USA; erica.woodahl@umontana.edu; 3 Southcentral Foundation, Anchorage, AK 99508, USA; RRobinson@SouthcentralFoundation.com; 4 United States Public Health Service, Department of Human Services, Washington, DC 20201, USA; 5 Center for Alaska Native Health Research, University of Alaska Fairbanks, Fairbanks, AK 99775, USA; bert.boyer@gmail.com; 6 Bioethics & Humanities, University of Washington, Seattle, WA 98195, USA; wburke@uw.edu

**Keywords:** American Indian, Alaska Native, First Nations, Inuit, Mexican Amerindians, cytochrome P450, pharmacogenetics, allele frequency, drug metabolism

## Abstract

Indigenous North American populations, including American Indian and Alaska Native peoples in the United States, the First Nations, Métis and Inuit peoples in Canada and Amerindians in Mexico, are historically under-represented in biomedical research, including genomic research on drug disposition and response. Without adequate representation in pharmacogenetic studies establishing genotype-phenotype relationships, Indigenous populations may not benefit fully from new innovations in precision medicine testing to tailor and improve the safety and efficacy of drug treatment, resulting in health care disparities. The purpose of this review is to summarize and evaluate what is currently known about cytochrome *P450* genetic variation in Indigenous populations in North America and to highlight the importance of including these groups in future pharmacogenetic studies for implementation of personalized drug therapy.

## 1. Introduction

Pharmacogenetics, a form of genomic medicine, aims to establish how genetic variation can affect an individual’s response to drugs, guiding the selection of the best drug and dose for a patient to improve healthcare quality [1]. The field of pharmacogenetics has the potential to improve health outcomes and reduce the cost of care by maximizing therapeutic success and minimizing the risk of adverse drug reactions or therapeutic failure at the population and potentially the individual level. However, a major issue in the translation of pharmacogenetic research into clinical practice is that existing databases have been populated from studies that lack significant ethnic and racial diversity.

Although diversity in genomic research, including pharmacogenetics, has increased in recent years, oversampling of populations of European ancestry continues to be a problem in the field. Indeed, the latest analysis of genome-wide association studies found that 81% of samples were from individuals of European ancestry [2]. The non-European portion was comprised of mostly Asian ancestry, leaving just 5% for the rest of the world’s populations [2]. Failure to include diverse populations in genomic studies leads to a biased understanding of the health implications of genetic variation and resulting medical findings may be preferentially beneficial to patients of European ancestry. Increased attention to genetic data from diverse populations is required to give “everyone the best chance at good health” [3]. This review article addresses the limited published pharmacogenetic research with Indigenous peoples of North America (Canada, United States and Mexico) and the challenges this poses for clinical practice. 

According to the 2011 National Household Survey in Canada, 1.4 million people reported Canadian Aboriginal identity, representing a population increase of 20% since 2006 [4]. Of this population, 60.8% identified as First Nations (FN) people, 32.3% as Métis and 4.2% as Inuit. We will refer to the individuals described in previous studies as Canadian Aboriginal, Canadian Native Indian and Canadian Indigenous as FN, Métis, or Inuit peoples, or more broadly as “Indigenous peoples of Canada,” in this review. The 2010 United States Census reported that 5.2 million American Indian and Alaska Native (AIAN) people live in the United States, with the AIAN population having grown 39% in the preceding decade [5]. The population of Mexico is stratified into two main groups: Amerindians, Indigenous people with over 68 ethnic groups representing 7% of the Mexican population and Mestizos, a group that arose as the result of admixture among Europeans, Amerindians and African slaves [6,7,8]. For the purposes of this paper, Amerindian will refer to Amerindian Indigenous populations from Mexico.

While much work has been conducted investigating the clinical importance of the cytochrome P450 (*P450*) gene variants, there is relatively little data specifically addressing *P450* variation and its consequences in Indigenous populations [9]. Geographical isolation, unbalanced resource allocation, failure of researchers to include Indigenous communities in study design and reluctance to participate in studies due to historical and recent research misconduct all contribute to under-representation of Indigenous populations in biomedical (including genetic) research studies [2,10,11,12,13,14]. However, it is important to understand the unique genetic variation that arises in these historically isolated populations because there are clinical implications of having uncharacterized genetic variation, particularly with drug metabolizing enzymes.

The *P450* genes encode a group of highly polymorphic enzymes that play a critical role in drug metabolism [15]. There are 57 *P450* genes in humans, with members of the CYP1, CYP2 and CYP3 families being responsible for most of the metabolic clearance of the approximately 75% of all drugs that are eliminated from blood by this process [16,17,18]. Variation in these *P450* genes can result in proteins with altered catalytic activity or abundance (referred to collectively hereafter as ‘enzyme activity’), leading to high inter-individual variability in systemic drug elimination and pharmacological response [19]. Gene sequence changes (single nucleotide variation and structural variation referred to collectively as alleles) that lead to altered P450 enzyme activity can be classified into four phenotypic groups: poor metabolizer (PM), intermediate metabolizer (IM), extensive metabolizer (EM) and ultra-rapid metabolizer (UM). PMs are generally homozygous for a variant allele that causes a complete loss of enzyme activity (null allele), IMs can be heterozygous for a reference allele and a null allele or a combination of reduced function alleles, EMs have two reference activity alleles and UMs have multiple copies of the *P450* gene or a variant that increases total enzyme activity, relative to the reference enzyme. Enzyme activity is inversely related to systemic parent drug exposure, which drives most pharmacological effects.

Indigenous populations can have distinct variant allele frequencies, which are related to historic geographical isolation and arise due to genetic drift, selective pressures and the founder effect. The population-level differences in *P450* allele frequencies in Indigenous peoples requires consideration to avoid negative clinical outcomes including the potential for phenotypic misclassification and inappropriate drug utilization, further contributing to health care disparities. This review focuses on what is currently known about *P450* pharmacogenetics in Indigenous North American populations and how the unique variation found in these populations may impact drug metabolism and response. We have intentionally clustered these peoples by their shared heritage and geographical proximity, though we acknowledge that each group has their own unique histories, languages and cultural traditions.

## 2. Methods

**Figure 1:**
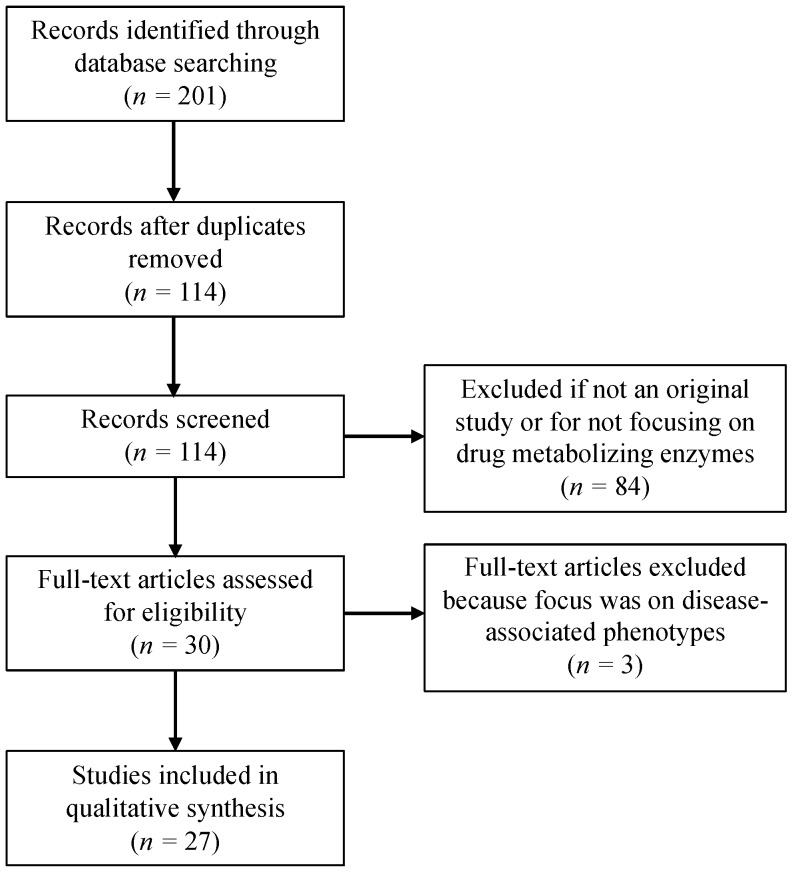
Flow diagram depicting the number of records identified, included and excluded in this review.

The primary focus of this review is to provide a summary of the *P450* pharmacogenetic research conducted with and for Indigenous North American populations. We conducted a systematic literature review to identify published studies of *P450* genetic variation, allele frequency and drug metabolism in AIAN, Indigenous peoples of Canada and Amerindians. A search of PubMed was performed using the keywords “Alaska Native,” “American Indian,” “Native American,” Canadian Native Indian,” “First Nations,” “Canadian Inuit,” “Mexico Amerindian,” “Mexico Indigenous,” “cytochrome P450 polymorphisms,” “Pharmacogenetics,” and “CYP450 allele frequencies,” “drug disposition,” and “drug metabolism.” Inclusion criteria were original research studies published in English and cited in PubMed between 1990 and October 2017. Figure 1 depicts the number of records identified and included or excluded by the aforementioned criteria. 

The following data were abstracted from selected studies: number of individuals in the study, the study population, P450 enzymes, method for genotyping and phenotyping, allele frequencies and conclusions from the study. For some of these studies, a reference population (e.g., European or Mestizos descent) was included. Our focus was to review available data for Indigenous North American populations, but we note that the studies reported in this review contain inconsistent approaches to population description for comparator populations, which may be categorized by race, ethnicity, nationality, or geographic location. Summary tables (Tables 2–13) also include reference data for different racial groups abstracted from the 1000 Genomes database (SNVs) [20] or from Zhou et al. [21] (complex haplotypes or structural variation not readily obtained from 1000 Genomes).

## 3. Results

**Figure 2:**
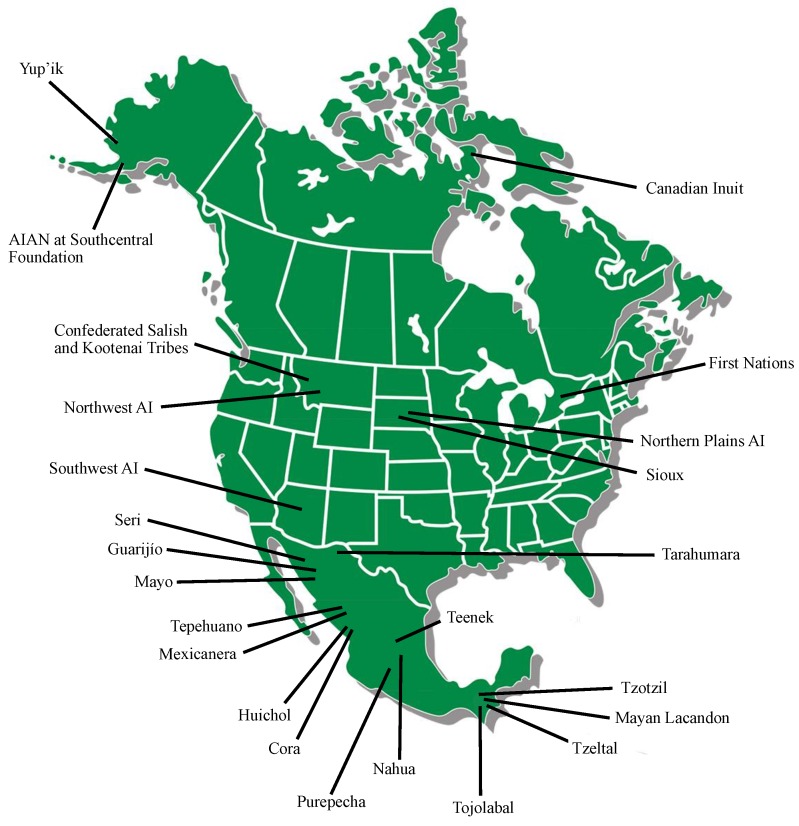
Map of North America with general locations of Indigenous populations included in this review.

We identified twenty-seven studies that met our inclusion criteria. These studies reported *P450* polymorphisms in Indigenous North American populations for *CYP1A1*, *CYP1A2*, *CYP2A6*, *CYP2B6*, *CYP2C9*, *CYP2C19*, *CYP2D6*, *CYP2E1*, *CYP3A4*, *CYP3A5* and *CYP4F2*. Six studies were in AIAN people, seven studies were in Indigenous people of Canada and fourteen studies were in Amerindian populations of Mexico. Figure 2 shows the geographical locations of the study populations and Table 1 summarizes the study results.

### 3.1. CYP1A1

**Figure 3:**
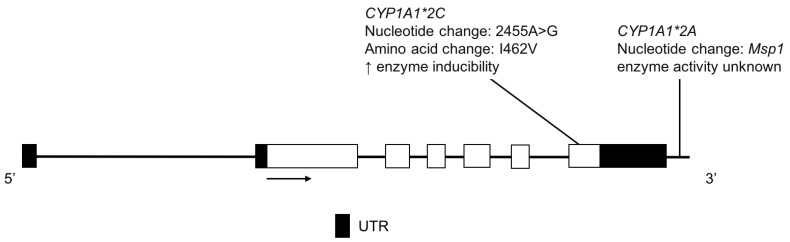
The human *CYP1A1* gene and variants detected in two Amerindian populations. Exon 1 is untranslated in *CYP1A1.* Open boxes represent exons, lines represent introns and shaded boxes represent untranslated region (UTR).

The CYP1A1 enzyme plays a role in the metabolism of caffeine [49] as well as the bioactivation of polycyclic aromatic hydrocarbons [50]. The CYP1A1*2A variant located in the 3′ non-coding region confers a restriction endonuclease site for cleavage by *Msp1* (Figure 3) [51]. CYP1A1*2C is characterized by a nonsynonymous base change that results in an amino acid substitution associated with an increase in *CYP1A1* gene inducibility (Figure 3) [52,53]. 

The frequencies of CYP1A1*2A and *2C were determined in two Amerindian peoples, the Teenek and Mayos and compared to the Mestizo Mexican population [22]. In the Teenek population, the minor allele frequencies (MAFs) of CYP1A1*2A and *2C were 71.4 and 65.4%, respectively and in the Mayos the MAFs were 46.9 and 54.6%, respectively (Table 2) [22]. Both Amerindian populations had a significantly higher frequency of CYP1A1*2C compared to the Mexican Mestizo population (34.4%), while only the Teenek had a significantly higher frequency of CYP1A1*2A compared to the Mestizo population (40.1%) [22]. 

### 3.2. CYP1A2

**Figure 4:**
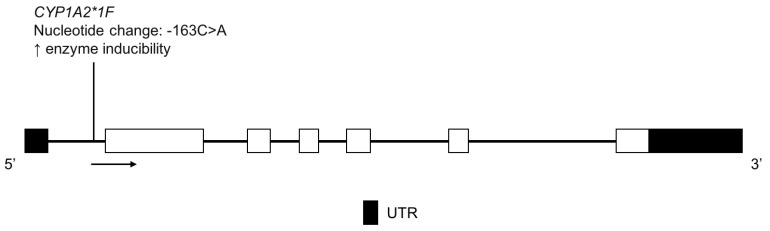
The human *CYP1A2* gene and the CYP1A2*1F variant detected in the Amerindian population. Exon 1 is untranslated in *CYP1A2.* Open boxes represent exons, lines represent introns and shaded boxes represent UTR.

CYP1A2 is a highly polymorphic enzyme responsible for the metabolism of many drugs including clozapine [54], mirtazapine [55], theophylline [56], tizanidine [57] and triamterene [58]. CYP1A2*1F is associated with increased enzyme activity in the presence of an inducer, such as high caffeine consumption or heavy cigarette use (Figure 4) [59,60,61]. 

De Andrés et al. found that the CYP1A2*1F allele was present at a MAF of 66.6% in the Amerindian population (Tarahumara, Tepehuano, Mexicanera, Huichol, Cora, Seri, Mayo and Guarijío) (Table 3) [23]. Using 100 mg of caffeine, as part of a probe drug cocktail for phenotyping, no association between CYP1A2*1F and higher enzyme activity was observed [23]. However, the CYP1A2*1F variant does not confer high constitutive activity but rather increases enzyme inducibility with exposure to an inducer, so the lack of genotype-phenotype association could be due to the fact that subjects were not stratified by their level of intake of a CYP1A2 inducer, such as high dose caffeine.

### 3.3. CYP2A6

**Figure 5:**
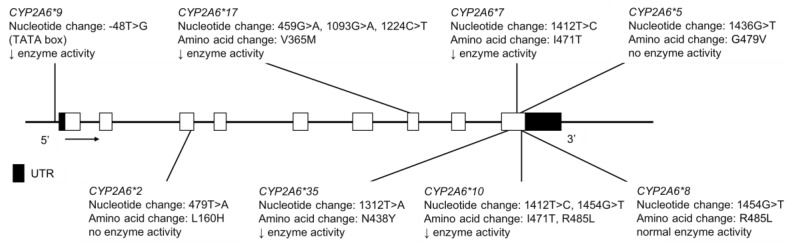
The human *CYP2A6* gene and variants tested for in AI, AN and FN populations. Not shown are CYP2A6*4, a full gene deletion and CYP2A6*12, an unequal crossover event where exons 1–2 are from *CYP2A7* and exons 3–9 from *CYP2A6* are merged. Open boxes represent exons, lines represent introns and shaded boxes represent UTR.

The CYP2A6 enzyme metabolizes some clinically used drugs as well as several pro-carcinogenic compounds. Its substrates include nicotine [62], tegafur [63], valproic acid [64,65], as well as the activity probe coumarin [66] and tobacco-related nitrosamines such as NNK [4-(methyl-nitrosamino)-1-(3-pyridyl)-1-butanone] [67] and NNN (*N*-nitrosonornicotine) [68]. Nicotine is one of the best studied CYP2A6 substrates. Renal clearance of nicotine is low and most of its metabolism (principally to cotinine) is catalyzed by CYP2A6; thus, associations between genetic variations in *CYP2A6* and nicotine metabolic clearance are strong [62,69]. In addition, CYP2A6 is the main enzyme responsible for converting cotinine to *trans*-3′-hydrocotinine [70,71,72]. This observation has led to use of the *trans*-3′-hydrocotinine/cotinine ratio (NMR) as a quantitative measure of CYP2A6 activity and nicotine exposure in cigarette smokers [73]. The decreased activity or loss of function variants, CYP2A6*2, *4, *5, *7, *9, *10, *12, *17 and *35 and several others, all reduce the rate of nicotine metabolism, compared to the reference allele (Figure 5) [69,73]. Moreover, *CYP2A6* genotype and the NMR have been associated with the efficacy of nicotine replacement therapy [74,75,76] and, following the success of a recent randomized clinical trial [77], have been proposed as biomarkers to guide drug selection for smoking cessation pharmacotherapy [78].

In the Yup’ik AN population, the MAFs for CYP2A6*2, *4, *9, *10 and *12 were found to be 0.4, 14.5, 8.9, 1.9 and 0.4%, respectively (Table 4) [24]. In the FN population, the MAFs for CYP2A6*4, *9 and *12 were 1.0, 15.5 and 0.5%, respectively and while CYP2A6*2 was not detected in this study, it was reported at a low MAF of 0.9% in a FN population by Nowak et al. (Table 4) [26,27]. These data highlight the very substantial differences in MAFs for a given P450 gene across indigenous populations.

Tanner et al. compared variation in *CYP2A6* and NMR in two different AI populations and assessed differences in relation to smoking behaviors and risks [25]. In Northern Plains (NP) AIs, the CYP2A6*2, *4, *9 and *12, the MAFs were 0.3%, 1.6%, 11.9% and 0.3%, respectively, while in AIs from the Southwest (SW) in Arizona the frequencies were 0.6, 0.3, 20.9 and 0.3%, respectively (Table 4) [25]. CYP2A6*7 and *17 were absent from the Yup’ik, NP and SW populations. While CYP2A6*35 was not found in the Yup’ik or NP, it had a MAF of 0.3% in the SW AI population (Table 4). The NP AI population had a lower frequency of *CYP2A6* decreased function alleles and a higher rate of nicotine metabolism, compared to SW smokers [25]. *CYP2A6* genetic variants are important to consider clinically because there are negative outcomes associated with higher rates of nicotine metabolism, including increased tobacco consumption, more difficulty with smoking cessation, poorer success with nicotine replacement therapy and elevated risk of lung cancer [27,75,79].

### 3.4. CYP2B6

**Figure 6:**
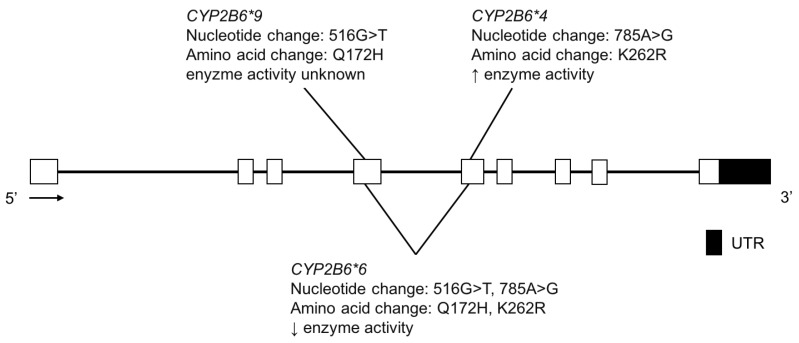
The human *CYP2B6* gene and variants tested for in the Yup’ik AN population. Open boxes represent exons, lines represent introns and shaded boxes represent UTR.

The CYP2B6 enzyme metabolizes bupropion [82,83], cyclophosphamide [84], efavirenz [85], ketamine [86,87], methadone [88], as well as other drugs. It is also thought to contribute to nicotine metabolism when CYP2A6 activity is low [89]. CYP2B6*4 (K262R) variation confers increased enzyme activity, while CYP2B6*6 is a haplotype that includes both K262R and Q172H and confers reduced function (Figure 6); it is common among different ethnic groups. For example, in the Yup’ik AN population, the reported CYP2B6*6, was 51.7% (Table 5) [24]. Unlike *CYP2A6*, *CYP2B6* genotype was not found to be associated with nicotine metabolism in this population. Previous studies found weak linkage disequilibrium between the *CYP2A6* and *CYP2B6* genes (localized together on chromosome 19), however, Binnington et al. reported strong linkage disequilibrium between these two genes in the Yup’ik population [24,90,91,92]. The authors proposed that the unique linkage disequilibrium observed between the reference CYP2A6*1B allele and the low activity CYP2B6*6 allele may be responsible for the higher nicotine metabolism in Yup’ik individuals with CYP2B6*6 genotype. 

### 3.5. CYP2C9

The CYP2C9 enzyme metabolizes medications across many therapeutic classes [93] including nonsteroidal anti-inflammatories (e.g., naproxen) [94,95,96,97], angiotensin II blockers (e.g., losartan) [98], as well as narrow therapeutic index drugs such as (*S*)-warfarin [94,99], tolbutamide [100] and phenytoin [101]. Warfarin dosing is challenging and regularly monitored due to its wide inter-individual variability and narrow therapeutic index, which affects both its pharmacokinetics and pharmacodynamic response. AN populations are reported to require a lower dose of warfarin to achieve a desired therapeutic effect, with the average daily dose for the AN population being 4.34 mg versus 5.19 mg for those of European descent [102]. This observed difference in warfarin dosing is clinically meaningful and thought to be due, in part, to genetic polymorphisms in the *CYP2C9, VKORC1* and *CYP4F2* genes [103]. For example, individuals with CYP2C9*2 or CYP2C9*3 variant alleles require a lower warfarin dose to achieve therapeutic anticoagulation [104]. 

In the Indigenous population of Canada, the previously studied *CYP2C9* variant allele frequencies were found to be distinct from the European and Asian reference groups, particularly for the Inuit population, where CYP2C9*2, *3 and *4 were absent [28]. The MAF of CYP2C9*2, *3 and *4 were 3.0%, 6.0% and 0.0%, respectively in the FN population (Table 6) [28].

Through deep resequencing to identify novel variants and subsequent genotyping to establish population frequencies, the prevalence of novel and previously known *CYP2C9* variants was determined in the Yup’ik AN, AIAN at Southcentral Foundation (SCF) (with multiple AN sub-cultures and an aggregate of AI tribes) and the Confederated Salish and Kootenai Tribes (CSKT) AI populations [32,33]. The MAFs of CYP2C9*2 and *3 were lower in AIAN populations with CYP2C9*2 at 0.3%, 5.2% and 5.2% in the Yup’ik, SCF and CSKT populations, respectively, compared to European populations at 15.2% (Table 6) [32,33]. The prevalence of CYP2C9*3 was found to be 2.1%, 3.4% and 2.7% in the Yup’ik, SCF and CSKT populations, respectively, compared to European populations at 6.6% (Table 6) [32,33]. CYP2C9*29, a rare coding-region variant, was found at 2.1% in the Yup’ik population [33]. In another study of AI youth in the Northwestern United States, the MAFs of CYP2C9*2, *3 and *5 were 5.8%, 2.7% and 0.4%, respectively (Table 6) [34]. 

**Figure 7:**
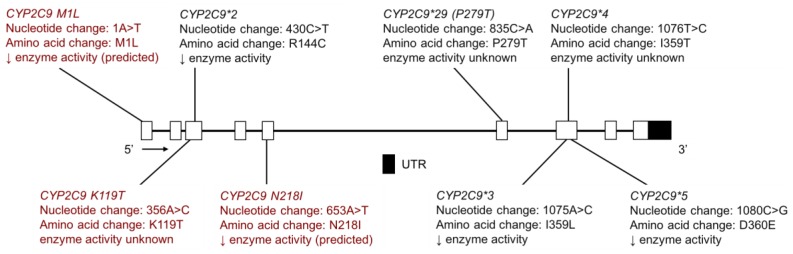
The human *CYP2C9* gene and single nucleotide variants (SNVs) tested for in AIAN, FN, Inuit and Amerindian populations. Highlighted in red are novel variants identified by resequencing *CYP2C9* in the Yup’ik, AIAN and Confederated Salish and Kootenai Tribes (CSKT) populations. Not shown is CYP2C9*6, a frameshift mutation that results in a shortened protein. Open boxes represent exons, lines represent introns and shaded boxes represent UTR.

With respect to novel variation, one new novel coding variant, *CYP2C9 K119T* (Figure 7), was identified in the CSKT population at a frequency of 0.57% [32]. In addition, two novel coding-region *CYP2C9* were identified in the Yup’ik and SCF populations: *CYP2C9 M1L* (*M1L*) and *CYP2C9 N218I (N218I)* (Figure 7) [33]. These two SNVs are of interest due to the fact that they are both coding variants and present at relatively high frequencies in the Yup’ik population; MAFs of *M1L* and *N218I* were 6.3% and 3.8%, respectively, whereas in the SCF population, the MAFs were 1.0% and 1.4%, respectively (Table 7). The switch from a methionine start codon to leucine for *M1L* is predicted to confer a PM phenotype in vivo for carriers of the *M1L* variant by severely slowing or stopping RNA translation and protein production. The *N218I* variant is also expected to have reduced enzyme activity as it had a Grantham score of 149 [33], where a score greater than 100 indicates that the amino acid substitution is predicted to be damaging. Presently, such in silico predictions cannot be relied upon and additional functional studies are needed [105]. However, there is increased confidence that individuals who are heterozygous or homozygous for the *M1L* variant would have a lower warfarin dose requirement, among phenotypic changes for other CYP2C9 substrates. 

Sosa-Macías et al. determined the CYP2C9*2, *3 and *6 allele frequencies in eight Amerindian populations from Northwest Mexico: Tepehuano, Mexicanera and Huichol from Durango, Cora from Nayarit, Seri, Guarijío and Mayo from Sonora and Tarahumara from Chihuahua [30]. The MAF of CYP2C9*2 in Seris and Mayos was 2.6% and 5.7%, respectively; this variant was not found in the other six Amerindian populations (Table 6) [30]. With regard to CYP2C9*3, the MAF was 10.4%, 9.1%, 7.5%, 6.7%, 3.7% and 3.3% in Tarahumaras, Mayos, Tepehuanos, Guarijíos, Coras and Huicholes, respectively, while it was not detected in Mexicanera or Seri Amerindians (Table 6) [30]. CYP2C9*6 was not detected in any of the eight Amerindian groups studied. Dorado et al. found that the MAF of CYP2C9*2 and *3 in the Tepehuano population was 1.0% and 1.5%, respectively, while CYP2C9*4, *5 and *6 were not detected (Table 6) [29]. Castelán-Martínez et al. tested for CYP2C9*2 and CYP2C9*3 in five Amerindian groups: Nahua from Central Mexico, Teenek from the Huasteca Potosina region, Tarahumara from Chihuahua, Purepecha from Michoacan and Huichol from Nayarit [31]. In the Nahua and Teenek groups, the CYP2C9*2 MAF was 0.7% and 0.5%, respectively and the CYP2C9*3 MAF was 0.4% and 0.5%, respectively (Table 6) [31]. The CYP2C9*2 and *3 alleles were absent from the Tarahumara, Purepecha and Huichol Amerindian groups [31]. 

With regard to CYP2C9*2 in the Tepehuano and Tarahumara populations, the results reported by Dorado et al. Sosa-Macías et al. and Castelán-Martínez et al. are in agreement, with this allele being absent or presenting at a low frequency, compared to Mestizos (6.9%) [29,30,31]. However, there is a notable difference in CYP2C9*3 allele frequencies reported by these authors for the Tepehuano and Tarahumara populations. Dorado et al. included 99 Tepehuanos, Castelán-Martínez et al. included 104 Tarahumaras and Sosa-Macías et al. included 127 Tepehuanos and 74 Tarahumaras. The small sample sizes may contribute to the observed difference in allele frequency. 

De Andrés et al. performed genotyping and phenotyping, using losartan as a probe substrate as part of a cocktail, in Amerindian population including the Tarahumara, Tepehuano, Mexicanera, Huichol, Cora, Seri, Mayo and Guarijío groups [23]. The ratio of losartan to losartan carboxylic acid was significantly greater in CYP2C9*2 or *3 homozygotes or carriers, compared to those with the reference genotype [23]. However, there were also three individuals whose high parent to metabolite could not be explained by their genotype, suggesting that there may be unidentified rare variants that confer PM phenotype in this Amerindian population [23]. 

### 3.6. CYP2C19

**Figure 8:**
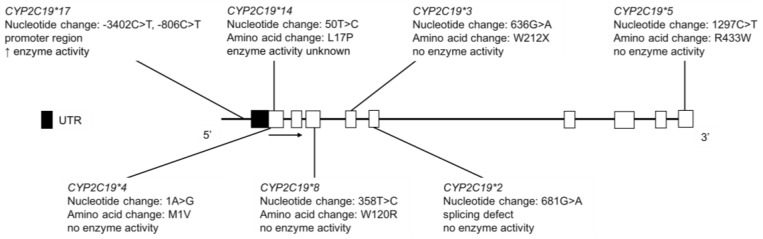
The human *CYP2C19* gene and SNVs genotyped for in AI, FN, Inuit and Amerindian populations. Open boxes represent exons, lines represent introns and shaded boxes represent UTR.

The CYP2C19 enzyme plays an important role in the metabolism of antiplatelet agents (e.g., clopidogrel) [106], proton pump inhibitors (e.g., omeprazole) [107], tricyclic antidepressants [108,109], selective serotonin reuptake inhibitors [110,111,112,113] and benzodiazepines [114]. CYP2C19*2 and *3 alleles define the current PM phenotype status (Figure 8), with markedly different allele frequencies across different ethnic populations. The CYP2C19*17 variant is a gain-of-function allele, resulting in higher enzyme activity (Figure 8) [115]. CYP2C19 catalyzes the 4′-hydroxylation of (S)-mephenytoin, a probe substrate for CYP2C19 [116,117]. The CYP2C19 PM phenotype is associated with a reduced ability to metabolize (*S*)-mephenytoin [118]. Thus, PMs eliminate racemic mephenytoin more slowly than EMs and urinary 4′-hydroxymephenytoin and *S*/*R* mephenytoin enantiomeric ratio was used in the past to distinguish between CYP2C19 EMs and PMs [119].

Nowak et al. reported that in FNs, the CYP2C19*2 allelic variant was found at 19.1%, while CYP2C19*3 was not detected (Table 8) [26]. Jurima-Romet et al. conducted *CYP2C19* genotyping and phenotyping, based on the mephenytoin S/R enantiomeric ratio, in Inuit peoples in Canada [35]. The CYP2C19*2 allele frequency was 12%, while CYP2C19*3 was not detected in this Inuit population (Table 8). Subjects ingested a single dose of 100 mg (*R*,*S*)-mephenytoin and collected urine for 12 h. Urinary S/R mephenytoin enantiomeric ratio was used to distinguish between EMs and PMs, where S/R ratio ≥ 1.0 indicated PM phenotype and S/R ratio ≤ 0.5 indicated EM phenotype. Genotype results were in agreement with phenotype results and as expected, individuals classified as PMs did not have detectable levels urinary 4′-hydroxymephenytoin [35].

Clopidogrel is a prodrug that is bioactivated by multiple P450s enzymes (including CYP2C19) to its active metabolite, which acts as an antiplatelet agent by irreversibly inhibiting the P2Y12 adenosine diphosphate receptor on platelets [106]. *CYP2C19* genetic variation has been associated with the fraction metabolized and clinical response to clopidogrel [120]. In the Oglala Sioux Tribe of South Dakota, the MAFs for CYP2C19*2, *3 and *17 were 11.2%, 0.0% and 8.7%, respectively (Table 8) [36]. P2Y12 reaction units (PRU) were used to evaluate the pharmacodynamic effect of clopidogrel, with lower values representing reduced platelet aggregation and higher efficacy of clopidogrel. Although PRU was not found to be significantly associated with genotype, the median PRU of 194 (range 29–400) is similar to values reported for other groups [36]. 

In a study examining *CYP2C19* variant alleles in AI children, the frequencies of CYP2C19*2, *4 and *17 were 11.5%, 1.3% and 11.1%, respectively (Table 8) [34]. CYP2C19*8 and CYP2C19*14 were not present in the AI population studied and CYP2C19*3 was not tested. Currently, nothing is known about genotype-drug disposition and response phenotype associations in AN populations.

With regards to *CYP2C19* variation in Amerindians of Mexico, Salazar-Flores et al. performed genotyping in Tarahumaras from Chihuahua, Purepechas from Michoacán, Tojolabales, Tzotziles and Tzeltales from Chiapas and Tepehuanos from Durango [37]. The CYP2C19*2 MAF was reported as 31%, 5.4%, 3.6%, 5.6% and 0.0% in Tarahumaras, Purepechas, Tojolabales, Tzotziles and Tzeltales, respectively (Table 8) [37]. The high allele frequency in the Tarahumara population is of particular interest clinically, as this predicts that a substantial portion of the population would be CYP2C19 PMs. CYP2C19*3, *4 and *5 were not detected in any of the Amerindian populations studied [37]. In an Amerindian population including the Tarahumara, Tepehuano, Mexicanera, Huichol, Cora, Seri, Mayo and Guarijío groups, de Andrés et al. reported the MAFs of CYP2C19*2, *3 and *17 as 12.0%, 0.2% and 2.2%, respectively (Table 8) [23]. CYP2C19*4 and *5 were not detected in the population studied [23]. CYP2C19 phenotype was determined using omeprazole as a probe substrate as part of a drug cocktail. The ratio of omeprazole to 5-hydroxyomeprazole was significantly greater in individuals with the PM conferring variants, CYP2C19*2 or *3, compared to those with the reference genotype [23]. There were some individuals whose genotype did not correspond to their phenotype, which the authors suggest may be due to population-specific alleles not identified in other populations [23]. 

### 3.7. CYP2D6

**Figure 9:**
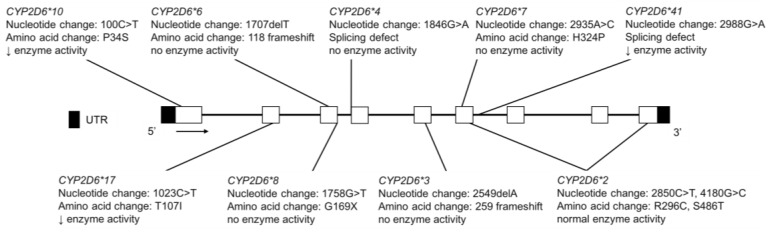
The human *CYP2D6* gene and variants tested for in the Inuit, FN, CSKT, AI youth and Amerindian populations. For variants that are part of a haplotype group, only the diagnostic SNVs commonly tested are shown. Not shown are copy number variations, CYP2D6*5, a full gene deletion and CYP2D6*35, which has normal enzyme activity and is the result of multiple nucleotide changes (−1584C>G, 31G>A, 1661G>C; 2850C>T and 4180G>C) and amino acid changes (V11M, R296C and S486T) in multiple exons. Open boxes represent exons, lines represent introns and shaded boxes represent UTR.

The CYP2D6 enzyme metabolizes many basic drugs including opioids [121,122], antidepressants (e.g., nortriptyline and fluoxetine) [108,111,123,124], antipsychotics (e.g., risperidone) [125,126] and ß-blockers (e.g., metoprolol) [127,128,129]. It is another example of a polymorphic enzyme with highly penetrant null (loss-of-function) allelic variants conferring a PM phenotype or copy number variation resulting in a UM phenotype. Individuals homozygous or compound heterozygous for the CYP2D6*3, *4, *5, or *6 variants exhibit no enzyme activity (Figure 9), while those with CYP2D6*1 or *2 gene duplication have more CYP2D6 protein with resulting higher enzymatic activity. This is clinically relevant for codeine, which is metabolized to morphine by CYP2D6. CYP2D6 PMs may experience an inadequate analgesic effect while UMs are at risk of morphine toxicity [130,131]. This is particularly important for the pediatric population for which codeine use was common and so in 2013 a US Food and Drug Administration Black Box Warning was issued for the drug. A structurally related drug, dextromethorphan (DEX), is *O*-demethylated by CYP2D6 to dextrorphan (DXO) and, therefore, the metabolic ratio of DEX/DXO can be used to differentiate between CYP2D6 phenotypes [132,133].

In Inuit peoples in Canada, the allele frequency of CYP2D6*4 was 6.7–8.3%; the CYP2D6*10 MAF was 2.2% and the CYP2D6*3 and *6 alleles were not detected (Table 9) [38]. Phenotyping, based on the urinary DEX/DXO metabolic ratio, was also performed. Study participants ingested a single dose of 30 mg dextromethorphan hydrobromide and collected urine overnight. Phenotype results were in agreement with genotype and furthermore, individuals classified as PMs had lower recoveries of DXO as well as other CYP2D6-mediated metabolites, compared to EMs. Notably, the frequency of the CYP2D6*4 allele in the Inuit population was significantly lower than the MAF reported in the European population (23%) [134,135] and significantly greater than in the Asian population (<1.0%) [136,137]. 

*CYP2D6* genotype, as well as phenotype using the *O*-demethylation ratio of DEX [138], was determined in a FN population [39]. The MAFs of CYP2D6*3, *4 and *10 were 0.0, 3.0 and 3.0%, respectively (Table 9) [39]. Interestingly, the one individual identified as a CYP2D6 PM by phenotyping with DEX was not found to have a CYP2D6*4/*4 genotype. This suggests that the PM phenotype could be attributed to a null allele not tested for in this study or a novel loss-of-function variant. 

In the CSKT population, the CYP2D6*1 frequency was 37.6%, CYP2D6*2 occurred at a frequency of 23.4% and copy number variation conferring UM phenotype had a frequency of 1.1% [32]. The reported frequencies of the PM conferring variants CYP2D6*3, *4, *5 and *6 were 0.3%, 20.9%, 1.3% and 0.0%, respectively (Table 9). The reduced activity variants conferring IM phenotype, CYP2D6*10, *17 and *41, had frequencies of 1.3%, 0.0% and 11.2%, respectively (Table 9). These allele frequencies are similar to the findings of McGrane and Loveland who performed pharmacogenetic testing in Northwest AI youth and reported MAFs of CYP2D6*3, *4, *5, *6, *10, *17 and *41 as 0.0%, 14.6%, 2.8%, 0.8%, 2.0%, 0.0% and 6.9%, respectively (Table 9) [34]. The relatively high frequencies for CYP2D6*4 and CYP2D6*41 could have clinical implications, particularly with medications that require bioactivation by CYP2D6 to an active metabolite to be clinically effective. Beyond codeine, another such drug is tamoxifen, an estrogen receptor antagonist used to prevent and treat estrogen-dependent breast cancer. CYP2D6 is the primary enzyme responsible for metabolizing tamoxifen to endoxifen, a major active metabolite responsible for much of tamoxifen’s therapeutic effect [139,140]. Therefore, it is important to understand the distribution of CYP2D6 activity within different patient populations. In the Northwest CSKT and AI youth populations, the overall prevalence of low activity CYP2D6, including both PMs and IMs, was 9.1% and 20.3%, respectively. 

Salazar-Flores et al. performed *CYP2D6* genotyping in Tarahumaras from Chihuahua, Purepechas from Michoacán, Tojolabales, Tzotziles and Tzeltales from Chiapas and Tepehuanos from Durango [37]. The MAFs reported for CYP2D6*4 were 7.3%, 2.9%, 1.2%, 2.7% and 5.3% for Tarahumaras, Purepechas, Tojolabales, Tzotziles and Tzeltales, respectively (Table 9) [37]. CYP2D6*3, *6, *7 and *8 were not detected in any of the Amerindian populations studied [37]. The *CYP2D6* genotype results from Perez-Paramo et al. in Tzotziles and Tzeltales, López-López et al. in Mayan Lacandones, Lazalde-Ramos et al. in Tarahumaras, Tepehuanos, Huicholes, Mexicaneros Coras, Seris, Guarijíos and Mayos, as well as two studies by Sosa-Macías et al. in Tepehuanos further support previous findings that the frequency of *CYP2D6* inactive alleles is low in most Amerindian populations (Table 9) [41,42,43]. These MAFs predict a low frequency of CYP2D6 PMs in these Amerindian populations. Sosa-Macías et al. and Lares-Asseff et al. investigated the CYP2D6 phenotype in Tepehuanos using DEX/DXO metabolic ratio and found that no Tepehuanos were classified as CYP2D6 PMs, as expected based on the allele frequencies reported by multiple studies [40,41]. The frequency of CYP2D6 UMs, based on CYP2D6*1 or *2 gene duplication, varied depending on the Amerindian population studied and the location of the reference Mestizo population [23,43,44,45].

The genotype results reported by de Andrés et al. in an Amerindian population including the Tarahumara, Tepehuano, Mexicanera, Huichol, Cora, Seri, Mayo and Guarijío groups, were consistent with previously published findings that *CYP2D6* variants conferring PM status are rare in the Amerindian population [23]. Regarding *CYP2D6* multiplications, the frequency of *wtxN*, *2xN and *4xN were 4.7, 1.1 and 0.1%, respectively [23]. CYP2D6 phenotype was also determined using the DEX/DXO ratio, with a significantly greater parent to metabolite ratio for individuals with reduced activity or null *CYP2D6* variants [23]. As with the other P450 drug metabolizing enzymes evaluated in this study, there was some discordance between genotype and phenotype. Further studies are necessary to identify and characterize variants that may impact the activity of important drug metabolizing enzymes including CYP2D6. 

### 3.8. CYP2E1

**Figure 10:**
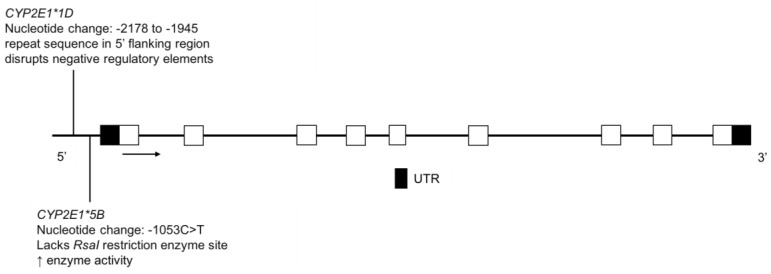
The human *CYP2E1* gene and the variants identified in the FN and Amerindian populations. Open boxes represent exons, lines represent introns and shaded boxes represent UTR.

The CYP2E1 enzyme metabolizes ethanol [141,142,143], tobacco-related nitrosamines [144], as well as other xenobiotics (e.g., acetaminophen) [145,146]. CYP2E1 is also induced by ethanol, increasing alcohol metabolism in cases of chronic ethanol consumption [146,147,148]. CYP2E1*1D has been associated with greater CYP2E1 induction by ethanol in individuals with at least one copy of the allele (Figure 10) [149]. The CYP2E1*5B allele, also referred to as CYP2E1*c2, has been associated with increased enzyme activity (Figure 10) [150,151].

The frequency of CYP2E1*1D was reported to be 9.3% in the FN population (Table 10), which is significantly higher than that seen in European Canadians (2.1%) [46]. Furthermore, FN individuals dependent on alcohol (as defined by DSM-IV) had a higher frequency of the CYP2E1*1D allele, compared to non-alcohol dependent FNs [46]. This same trend was found in Europeans and Southeast Asians [46]. CYP2E1*1D genotype was also associated with nicotine dependence in FNs, however further studies are needed to elucidate potential mechanisms responsible for this relationship [46].

The MAF of the *CYP2E1* −1295G>C variant was determined to be 51.5% in Huichols, an Amerindian population of Western-Central Mexico (Table 10) [47]. This frequency is very high compared to the Mexicans from Western Mexico (16.1%) and Europeans (1.7%) [47,152]. The *CYP2E1* −1295G>C variant is of interest when considering the metabolism of ethanol, as well as other CYP2E1 substrates and further studies would be useful to establish the clinical relevance of CYP2E1 variation across diverse populations.

### 3.9. CYP3A4 and CYP3A5

**Figure 11:**
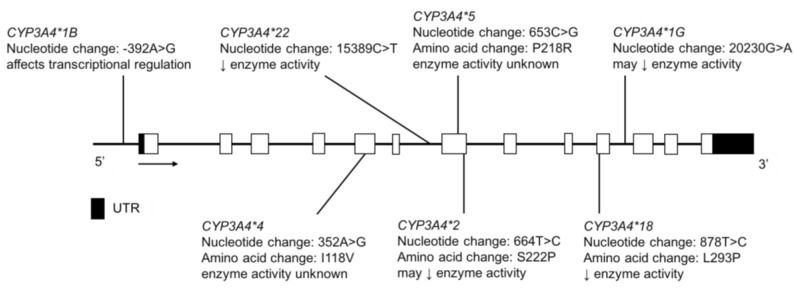
The human *CYP3A4* gene and SNVs tested for in the CSKT and Amerindian populations. Novel non-coding variants are not shown. Open boxes represent exons, lines represent introns and shaded boxes represent UTR.

**Figure 12:**
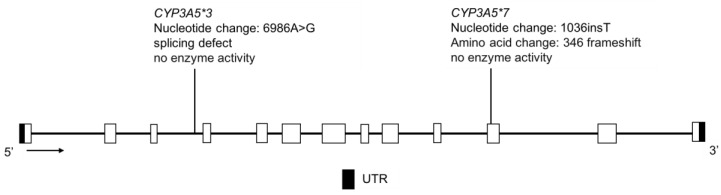
The human *CYP3A5* gene and the variants tested for in the CSKT population. Not shown is CYP3A5*6, where alternative splicing results in exon 7 skipping. Open boxes represent exons, lines represent introns and shaded boxes represent UTR.

The CYP3A4 and CYP3A5 enzymes have overlapping substrate specificity and together, they control the clearance of approximately 50% of all drugs eliminated primarily through P450-mediated biotransformation [15]. CYP3A4 protein is present in almost all adults, whereas CYP3A5 protein expression varies across different ethnic groups [153]; polymorphisms in the genes encoding these proteins are shown in Figure 11 and Figure 12. The CYP3A5 enzyme is expressed in individuals having at least one CYP3A5*1 allele, while those with two inactive alleles, CYP3A5*3, *6, or *7, encode a nonfunctional protein (Figure 12); the PM phenotype is most common in Europeans and less so in Asians and African Americans [154,155]. The CYP3A4*22 variant is also associated with reduced CYP3A4 protein levels and enzyme function (Figure 11) [156,157,158], as are rare deleterious coding variants [159]. The CYP3A4*1B and CYP3A4*1G alleles (Figure 11) reportedly affect gene transcription but functional effects are unclear, as the data are mixed and interpretation is complicated by high linkage disequilibrium with CYP3A5*1 [160,161,162].

In the CSKT population, resequencing followed by subsequent genotyping identified four novel *CYP3A4* SNVs—three intronic and one in the 5’ UTR region. The known *CYP3A4* variants, *1B, *22 and *1G were found at frequencies of 2.2%, 2.4% and 26.8%, respectively (Table 11) [32]. This combination of allele frequencies may result in haplotypes conferring altered enzyme activity, which remains to be tested. With regard to *CYP3A5* in the CSKT population, CYP3A5*1 was detected at a frequency of 7.5%, CYP3A5*3 at 92.5%, while CYP3A5*6 and *7 were not detected (Table 12). These data suggest that 14.9% of CSKT individuals express CYP3A5, contributing to their total CYP3A metabolic activity. 

Reyes-Hernández et al. determined the MAFs of *CYP3A4* variants in the Tepehuano and Mestizo populations and found that the frequencies were not significantly different between these two group [48]. The MAFs of CYP3A4*1B was 8.0% in Tepehuanos, compared to 8.8% in Mestizos and CYP3A4*2 was not detected in the Tepehuanos but was found at a low frequency of 0.5% in Mestizos (Table 11) [48]. CYP3A4*4, *5 and *18 were not found in either population [48]. Although the variant allele frequencies were similar between Tepehuanos and Mestizos for *CYP3A4*, it is important to consider that these populations may share similar allele frequencies in other *P450* genes. De Andrés et al. found that the CYP3A4*1B MAF was 4.8% in an Amerindian population including the Tarahumara, Tepehuano, Mexicanera, Huichol, Cora, Seri, Mayo and Guarijío groups (Table 11) [23]. The CYP3A4*1B allele did not significantly affect the parent to metabolite ratio of dextromethorphan to 3-methoxymorphinan [23], a CYP3A4 mediated pathway [163].

### 3.10. CYP4F2

**Figure 13:**
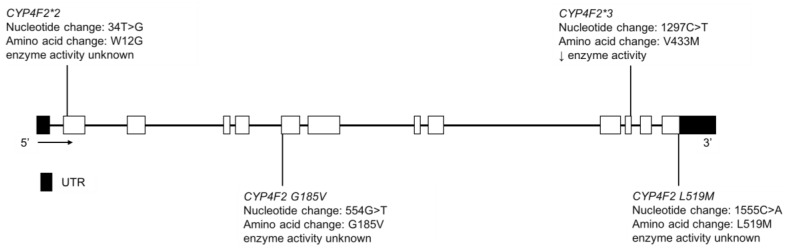
The human *CYP4F2* gene and SNVs detected in the Yup’ik and SCF populations. Not shown is *CYP4F2 spliceCG*, a novel splice variant that changed the splice site of exon 1. Exon 1 is untranslated in *CYP4F2.* Open boxes represent exons, lines represent introns and shaded boxes represent UTR.

CYP4F2 enzyme catabolizes vitamin K and, along with CYP2C9 and vitamin K oxidoreductase (VKOR), can affect the pharmacological response of warfarin, a VKOR antagonist. The Yup’ik AN population has a high frequency of CYP4F2*3 (Figure 13), which is a vitamin K sparing variant [164] and is associated with a higher warfarin dose requirement [165]. It is found at a frequency in the Yup’ik AN population higher than that seen elsewhere in the world, with one exception [166]. And is associated with a relatively high hepatic vitamin K status [33,167]. Selective pressure may have acted on the *CYP4F2* gene in the Yup’ik population to conserve vitamin K due to the inconsistent access to tundra greens throughout the year [33,167,168]. The MAF of CYP4F2*3 is reported to be 50.9% and 31.5% in the Yup’ik and SCF populations, respectively (Table 13) [33]. While the contribution of the *CYP4F2* variation to warfarin dose requirement is relatively low in European or African American populations, it may take on greater significance in AN populations due to the higher frequency of the CYP4F2*3 variant. 

Fohner et al. also reported the MAFs of CYP4F2*2, *L519M*, *G185V* and *spliceCG* in the Yup’ik population as 3.7%, 0.0%, 0.3% and 0.7%, respectively, while in the AIAN cohort at SCF they were 11.0%, 2.7%, 2.2% and 1.4%, respectively (Table 13) [33]. The functional impact of these variants is unclear but some may be deleterious.

## 4. Discussion

Pharmacogenetics is a growing field that presents the opportunity to improve safety and clinical outcomes of currently available treatments using individual genomic data. The implementation of this practice in all populations requires the elucidation and comprehensive understanding of genetic variation and its impact on drug phenotypes. In order to optimize clinical therapy and minimize adverse drug events in underserved Indigenous populations, these groups must be adequately represented in pharmacogenetic studies that identify genetic variation and inform on the dosing drugs that have established clinical associations with pharmacogene variation (e.g., abacavir, 6-mercaptopurine, warfarin, codeine), especially with narrow therapeutic index treatments. 

Not only do Indigenous groups often have different allele frequencies compared to other global populations but marked differences in allele frequencies can also be found between subcultures within a given geographical region. Notable findings from the original studies highlighted in this review include the lower frequency of *CYP2A6* loss-of-function alleles and a higher rate of nicotine metabolism in the NP AI, compared to a population of SW AI smokers [25]. With regards to *CYP2C9*, there was a lack of CYP2C9*2 in the Canadian Inuit population as well as many Amerindian groups including the Mexicanera, Huichol, Guarijío, Cora, Tarahumara and Purepecha [28,30,31]. CYP2C9*3 was also absent from the Canadian Inuit, Mexicanera, Seri, Purepecha and Huichol populations [28,30,31]. In contrast, CYP2C19*2 was found at a high MAF in Tarahumaras, compared to other Indigenous peoples of Mexico [37]. The relatively high frequencies for CYP2D6*4 and CYP2D6*41 in the CSKT and AI youth may be important to consider with CYP2D6 substrates such as codeine, tamoxifen and antidepressants [32,34]. Conversely, certain subgroups in the Amerindian population (Tepehuano, Purepecha, Tojolabal, Tzotzil, Mexicanera, Cora and Guarijío) had a low proportion of *CYP2D6* variants conferring PM status [37,41,42,43]. About 15% of the CSKT population would be expected to express CYP3A5, based on the CYP3A5*1 allele frequency [32]. In the Yup’ik AN population, the CYP4F2*3 variant is expressed at a frequency of 50.9%, one of the highest MAFs seen across global populations for this SNV [33]. 

Indigenous populations of North America may also have novel *P450* gene variation not seen in other populations of the world that can potentially influence drug phenotype. The allele frequencies of known and recently reported *CYP2C9* variants in the AN Yup’ik population illustrate this scenario. The CYP2C9*2 and *3 alleles that define the CYP2C9 PM phenotype in the European population are found at very low frequencies in the Yup’ik population. The novel and relatively common *M1L* and *N218I* variants found in the Yup’ik population, in addition to CYP2C9*2 and *3 alleles, that are predicted to confer a CYP2C9 PM phenotype. Importantly, if only the allele frequencies known to be clinically relevant in the European population are applied to the Yup’ik population, an individual homozygous or heterozygous for the *M1L* or *N218I* variants would be classified as a CYP2C9 EM and improper warfarin dosing could result in adverse events for these individuals. This potential for misclassification and inappropriate drug dosing has also been described of African populations, where the CYP2C9*8 allele contributes to the PM phenotype [169]. This was also suggested by the work of de Andrés et al. where the CYP2C9, CYP2C19, or CYP2D6 phenotype for some Amerindians could not be accurately predicted based on genotype, possibly due to the presence of novel rare variation in these pharmacogenes [23].

Further studies are needed to identify and establish the allele frequencies of both known and novel variants in Indigenous populations, particularly in all *P450* genes that encode enzymes that have a clinically significant impact on drug disposition. For example, despite the fact that CYP1A2 is a highly polymorphic enzyme important for the metabolism of many clinical drugs, no studies to date have assessed the frequencies of *CYP1A2* allelic variants in Indigenous peoples of Canada or AIAN populations. Moving forward, it will also be important to improve genotyping and sequencing quality as well as increase study sample size, as these will help improve imputation and haplotype estimation in Indigenous populations, which may lead to the discovery of additional P450 SNVs or structural variants. Furthermore, there are problematic inconsistencies in population description for genetic studies, which is a recognized problem in the field [170]. In addition, the studies reported in this review do not fully capture the diversity of AI and other Indigenous tribes in North America. As seen in Figure 1, we found no studies from the US that were east of the Mississippi, leaving significant uncertainty about P450 genetic diversity for these people and drug phenotype relationships. In 2008, Jaja et al. conducted a systemic review of *P450* variation in Indigenous and Native American Populations that identified ten original studies, of which six of were from Canada, four from North, Central and South America and none in AIAN [9]. This review identified twenty-seven original studies, with six in AIAN, seven in Indigenous people of Canada and fourteen in Amerindian populations of Mexico.

One method for increasing representation of Indigenous people in genetic studies is first to form collaborative research partnerships, in which community partners share control of the research process and apply the values and procedures of community-based participatory research to establish research priorities and acceptable conditions under which the research will occur [10]. We formed a research network involving three tribal organizations and three universities in 2010, to address the dearth of information about pharmacogenomics in AIAN populations. The research network built on several years of research and partnership development at three research sites. (1) Investigators at the Center for Alaska Native Health Research had established research partnerships with the Yukon-Kuskokwim Health Corporation, serving 23,000 Yup’ik people in southwestern Alaska and with several communities in the Yukon-Kuskokwim River Delta [171]. (2) SCF, a tribally owned and operated healthcare organization had established a Research Department and developed collaborative projects with investigators from the University of Washington. SCF is based in Anchorage and provides healthcare services to 65,000 AIAN customer-owners, serving about 55% of the total AN population in Alaska [171,172]. (3) Investigators at University of Montana had established partnerships with the CSKT of the Flathead Indian Reservation in northwestern Montana to pursue research with the Bitterroot Salish, Upper Pend d’Oreille and Kootenai tribes. There are >7900 enrolled CSKT members, with a large number of descendants [171,173]. These research partnerships provided the foundation for the Northwest-Alaska Pharmacogenomic Research Network, academic-tribal partnerships initiated with support from the National Institutes of Health. This review highlights findings of research derived from collaborations we have initiated, as well as additional relevant publications identified in the literature search. 

The limited data on *P450* genetic variation in Indigenous North American populations translates to missed opportunities for optimizing care. Interestingly, the data that does exist for Indigenous North American people suggests that they have unique genetic variation profiles that may critically impact their response to drug therapy. Without a complete understanding of this population’s unique pharmacogene variation profile, Indigenous people may not derive the same benefit from genomics-based precision medicine as the European population. The populations included in pharmacogenetic research stand to gain the most from clinical trials findings that establish test validity and utility. A better understanding of the unique *P450* pharmacogenetic variation in Indigenous populations is needed if these communities are to be included in clinical decisions regarding personalized drug therapy and policies surrounding precision medicine. 

## Figures and Tables

**Table 1:** Studies of *P450* genetic polymorphisms in Indigenous North American populations.

Reference	Genes	Population (Tribal Group or Affiliation)	Genotyping/Phenotyping Method and Study Conclusion
Fragoso, 2005 [22]	*CYP1A1*	106 Amerindian (Teenek and Mayo)	Genotype was determined by allele-specific PCR. The frequency of *CYP1A1* variants is distinct for Amerindian and Mestizo populations of Mexico.
de Andrés, 2017 [23]	*CYP1A2* *CYP2C9* *CYP2C19* *CYP2D6* *CYP3A4*	450 Amerindian (Tarahumara, Tepehuano, Mexicanera, Huichol, Cora, Seri, Mayo and Guarijío)	Genotype was determined by RT-PCR and then compared to phenotype, which was determined by a probe substrate cocktail approach using caffeine for *CYP1A2,* losartan for *CYP2C9,* omeprazole for *CYP2C19*, followed by dextromethorphan for *CYP2D6* and *CYP3A4*. Further studies are needed to identify and characterize rare variants in the Amerindian population to improve genotype-phenotype predictions.
Binnington, 2012 [24]	*CYP2A6* *CYP2B6*	400 AN (Yup’ik)	Genotype was determined by two-step allele-specific PCR. Study found an association between nicotine metabolism and *CYP2A6* genotype. High CYP2A6 activity may contribute to the high risk of tobacco-related diseases in the Yup’ik AN population.
Tanner, 2017 [25]	*CYP2A6*	636 AI (Northern Plains and Southwest tribes)	Genotype was determined by two-step allele-specific PCR and RT-PCR. The Northern Plains and Southwest AI populations have unique profiles of *CYP2A6* genetic variation that contributes to differences in nicotine metabolism and tobacco-related disease risks.
Nowak, 1998 [26]	*CYP2A6* *CYP2C19*	159 FN	Genotype was determined by PCR-RFLP. FN people have distinct frequencies of variant alleles in *CYP2A6* and *CYP2C19* compared to European and Asian populations.
Schoedel, 2004 [27]	*CYP2A6*	101 FN	Genotype was determined by two-step allele-specific PCR. *CYP2A6* allele frequencies were markedly different between FN and other ethnic groups, suggesting differences in nicotine metabolism.
Gaedigk, 2001 [28]	*CYP2C9*	153 FN 151 Inuit	Genotype was determined by PCR-RFLP. The *CYP2C9* allele frequencies in the FN and Inuit populations differ from the European and Asian reference populations, likely as a result of genetic drift and selective pressures
Dorado, 2011 [29]	*CYP2C9*	99 Amerindian (Tepehuano)	Genotype was determined by RT-PCR. *CYP2C9* variation in Tepehuanos and Mestizos was found to be distinct compared to that reported in Mexican Americans and Spaniards
Sosa-Macías, 2013 [30]	*CYP2C9*	505 Amerindian (Tepehuano, Mexicanera, Huichol, Seri, Guarijío, Mayo, Cora and Tarahumara)	Genotype was determined by RT-PCR. The allele frequencies for *CYP2C9* variants that confer PM phenotype were determined in eight Amerindian groups of Northwest Mexico.
Castelán-Martínez, 2013 [31]	*CYP2C9*	483 Amerindian (Nahua, Teenek, Tarahumara, Purepecha and Huichol)	Genotype was determined by RT-PCR. Two PM conferring *CYP2C9* variants were investigated in five Amerindian populations and compared to other reports of *CYP2C9* variation in Amerindians and Mestizos.
Fohner, 2013 [32]	*CYP2C9* *CYP2D6* *CYP3A4* *CYP3A5*	94–187 AI (Salish, Pend d’Oreille and Kootenai)	*CYP2D6* was completely resequenced, while exons, adjacent introns and flanking regions were resequenced for *CYP3A4*, *CYP3A5* and *CYP2C9*. Sanger sequencing was used for resequencing and *CYP2D6* copy number was determined by PCR. Findings from pharmacogenetic studies conducted in European populations do not necessarily apply to AIAN populations. Particularly with *CYP3A4* allele frequency, the Confederated Salish and Kootenai Tribes have unique allelic variation distinct from European Americans.
Fohner, 2015 [33]	*CYP2C9* *CYP4F2*	380 AIAN (multiple AN sub-cultures and aggregate of AI tribes) 350 AN (Yup’ik)	Allele frequencies of novel and previously known variants in warfarin pharmacogenes were determined by Sanger resequencing, followed by targeting genotyping, using the Fluidigm platform, in AN and AI populations.
McGrane and Loveland, 2016 [34]	*CYP2C9* *CYP2C19* *CYP2D6*	123 AI (Northwest)	Genotype was determined by qPCR. Study identified differences in genetic polymorphism frequencies in AI and European American youth in the US Northwest.
Jurima-Romet, 1996 [35]	*CYP2C19*	155 Inuit	Genotype results for *CYP2C19,* determined by allele-specific PCR, were found to be concordant with phenotype results, using R/S mephenytoin enantiomeric ratio. The *CYP2C19* PM variant allele frequencies in the Inuit population appear to be more similar to the European, rather than Asian, population.
Oestreich 2014, [36]	*CYP2C19*	100 AI (Sioux)	Genotype was determined by RT-PCR. The prevalence of *CYP2C19* PM conferring variants was determined to be lower or similar to Europeans. No significant association was observed between genotype and a marker for clopidogrel effectiveness.
Salazar-Flores, 2012 [37]	*CYP2C19* *CYP2D6*	365 Amerindian (Tarahumara, Purepecha, Tojolabal, Tzotzil and Tzeltal)	Genotype was determined by SNapShot multiplex PCR. With the exception of the Tarahumaras, the frequency of *CYP2C19* variants that confer PM phenotype was low. The frequency of CYP2D6 PMs is also expected to be low in these Amerindian populations.
Jurima-Romet, 1997 [38]	*CYP2D6*	155 Inuit	Genotype was determined by PCR-RFLP and dextromethorphan was used as a probe for CYP2D6 phenotype. Genotype results for *CYP2D6* were found to be concordant with phenotype results. The Inuit population had unique *CYP2D6* variation, distinct from European or Asian populations.
Nowak, 1997 [39]	*CYP2D6*	156 FN	Genotype was determined by mutation-specific PCR and dextromethorphan was used as a probe for CYP2D6 phenotype. The FN population had a low frequency of *CYP2D6* variants that result in decreased metabolic activity, compared to European and Asian populations.
Lares-Asseff, 2005 [40]	*CYP2D6*	55 Amerindian (Tepehuano)	All Tepehuanos included in this study were found to be CYP2D6 EMs by phenotyping with dextromethorphan.
Sosa-Macías, 2006 [41]	*CYP2D6*	101 Amerindian (Tepehuano)	Genotype was determined by PCR-RFLP and dextromethorphan was used as a probe for CYP2D6 phenotype. The distribution of *CYP2D6* variant alleles was markedly different between Tepehuano Amerindians and Mestizos; no Tepehuanos were classified as CYP2D6 PMs.
Sosa-Macías, 2010 [42]	*CYP2D6*	99 Amerindian (Tepehuano)	This study expanded upon Sosa-Macías et al. 2006 by genotyping for additional *CYP2D6* variants, which had different frequencies than that observed in Mestizos. RT-PCR and XL-PCR were used to determine genotype.
Lazalde-Ramos, 2014 [43]	*CYP2D6*	508 Amerindian (Tarahumara, Tepehuano, Huichol, Mexicanera, Cora, Seri, Guarijío and Mayo)	Genotype was determined by XL-PCR and copy number was evaluated by RT-PCR. The Amerindian populations included in this study had a lower frequency of *CYP2D6* PM conferring variants but a higher frequency of gene duplication conferring UM phenotype, compared to the Mestizo population.
López-López, 2014 [44]	*CYP2D6*	154 Amerindian (Mayan Lacandon)	Genotype was determined by XL-PCR. The Amerindian population had a low frequency of *CYP2D6* low or null activity alleles, compared to Mestizos. The frequency UM genotypes, determined by PCR-RFLP, were similar between Mayan Lacandones and Mestizos.
Perez-Paramo, 2015 [45]	*CYP2D6*	110 Amerindian (Tzotzil and Tzeltal)	Genotype was determined by XL-PCR. The *CYP2D6* alleles that confer low or null activity, as well as the gene duplication that confers UM phenotype, had lower frequencies in the Amerindian population compared to the Mestizo population.
Howard, 2003 [46]	*CYP2E1*	114 FN	PCR based genotyping and size discrimination by agarose gel were used to determine genotype. Compared to Canadian Europeans, the FN population had a significantly higher frequency of a *CYP2E1* variant associated with greater enzyme induction.
Gordillo-Bastidas, 2010 [47]	*CYP2E1*	101 Amerindian (Huichol)	Genotype was determined by PCR-RFLP. Compared to other world populations, the Huichol population had a high frequency of a *CYP2E1* variant associated with higher enzyme activity.
Reyes-Hernández, 2008 [48]	*CYP3A4*	100 Amerindian (Tepehuano)	Genotype was determined by PCR-RFLP. *CYP3A4* variation was not significantly different between the Tepehuano and Mestizo populations.

PCR: Polymerase Chain Reaction; RT-PCR: real-time PCR; AN: Alaska Native; FN: First Nations; RFLP: restriction fragment length polymorphism; qPCR: quantitative Polymerase Chain Reaction; AI: American Indian; PM: poor metabolizer; UM: ultra-rapid metabolizer; XL-PCR: EXtra Long Polymerase Chain Reaction.

**Table 2:** Comparison of *CYP1A1* allele frequencies in Indigenous North American populations to global populations from the 1000 Genomes Project [20]. *N* represents the number of alleles. Global populations are abbreviated as follows: African Ancestry in Southwest US (ASW); Utah residents with Northern and Western European ancestry (CEU); Han Chinese in Beijing, China (CHB); Mexican Ancestry in Los Angeles, California (MXL).

Country	Population	*N*	*CYP1A1* MAF (%)		Refs.
			*2A rs4646903	*2C rs1048943	
Mexico	Mayo	108	46.9	54.6	[22]
	Teenek	104	71.4	65.4	[22]
Multiple Countries (1000 Genomes)	MXL	128	39.8	33.6	[20]
	CHB	206	43.7	26.7	[20]
	CEU	198	9.1	4.0	[20]
	ASW	122	27.1	5.7	[20]

MAF: minor allele frequency.

**Table 3:** Comparison of *CYP1A2* allele frequencies in Indigenous North American populations to global populations from the 1000 Genomes Project [20]. *N* represents the number of alleles. Global populations are abbreviated as follows: African Ancestry in Southwest US (ASW); Utah residents with Northern and Western European ancestry (CEU); Han Chinese in Beijing, China (CHB); Mexican Ancestry in Los Angeles, California (MXL).

Country	Population	*N*	CYP1A2*1F MAF (%)	Refs.
			rs762551	
Mexico	Aggregate of Amerindian tribes (Tarahumara, Tepehuano, Mexicanera, Huichol, Cora, Seri, Mayo and Guarijío)	896	66.6	[23]
Multiple Countries (1000 Genomes)	MXL	128	73.4	[20]
	CHB	206	63.6	[20]
	CEU	198	72.7	[20]
	ASW	122	63.9	[20]

**Table 4:** Comparison of *CYP2A6* allele frequencies in Indigenous North American populations to global populations from the 1000 Genomes Project [20]. *N* represents the number of alleles. Global populations are abbreviated as follows: African Ancestry in Southwest US (ASW); Utah residents with Northern and Western European ancestry (CEU); Han Chinese in Beijing, China (CHB); Mexican Ancestry in Los Angeles, California (MXL). For alleles not captured by 1000 Genomes (noted by ‡), the frequencies were extracted from Exome Aggregation Consortium [80] and for alleles defined by multiple variants, the frequencies reported in Zhou et al. [21] using LDLink software [81] were used. Global population data for CYP2A6*35 was not included because it is currently difficult to accurately genotype due to the high homology to *CYP2A7*, which can result in false positives and false negatives.

Country	Population	*N*	*CYP2A6* MAF (%)										Refs.
			*2 rs1801272	*4	*5 rs5031017	*7 rs5031016	*8 rs28399468	*9 rs28399433	*10 rs5031016, rs28399468	*12	*17 rs28399454	*35 rs143731390	
Canada	FN	432	0.9	-	-	-	-	-	-	-	-	-	[26]
	FN	202	0.0	1.0	0.5	0.0	0.0	15.5	0.0	0.5	-	-	[27]
USA	Yup’ik	722	0.4	14.5	-	0.0	0.0	8.9	1.9	0.4	0.0	0.0	[24]
	NP AI	636	0.3	1.6	-	0.0	-	11.9	-	0.3	0.0	0.0	[25]
	SW AI	344	0.6	0.3	-	0.0	-	20.9	-	0.3	0.0	0.3	[25]
Multiple Countries (1000 Genomes)	MXL	128	1.6	-	<0.1 ^‡^	0.3 ^‡^	-	10.2	-	-	0.0	-	[20]
	Latino ^‡^	11,576 ^‡^											[80] ^‡^
	CHB	206	0.0	17 ^‡^	0.1 ^‡^	12.9 ^‡^	0.3 ^‡^	26.7	0.3 ^‡^	-	0.0	-	[20]
	East Asian ^‡^	8528 ^‡^											[21,80] ^‡^
	CEU	198	3.5	1.0 ^‡^	<0.1 ^‡^	0.2 ^‡^	0.3 ^‡^	5.1	<0.1 ^‡^	-	0.0	-	[20]
	European ^‡^	66,714 ^‡^											[21,80] ^‡^
	ASW	122	0.8	1.5 ^‡^	<0.1 ^‡^	0.0 ^‡^	0.3 ^‡^	10.7	<0.1 ^‡^	-	7.4	-	[20]
	African ^‡^	10,404 ^‡^											[21,80] ^‡^

**Table 5:** Comparison of *CYP2B6* allele frequencies in an Indigenous North American population to global populations from the 1000 Genomes Project [20]. *N* represents the number of alleles. Global populations are abbreviated as follows: African Ancestry in Southwest US (ASW); Utah residents with Northern and Western European ancestry (CEU); Han Chinese in Beijing, China (CHB); Mexican Ancestry in Los Angeles, California (MXL). For alleles not captured by 1000 Genomes (noted by ‡), the frequencies were extracted from Exome Aggregation Consortium [80] and for alleles defined by multiple variants, the frequencies reported in Zhou et al. [21] using LDLink software [81] were used.

Country	Population	*N*	*CYP2B6* MAF (%)			Refs.
			*4 rs2279343	*6 rs2279343, rs3745274	*9 rs3745274	
USA	Yup’ik	722	0.0	51.7	0.0	[24]
Multiple Countries (1000 Genomes)	MXL	128	3.4 ^‡^	-	31.3	[20]
	Latino ^‡^	10,418 ^‡^				[80] ^‡^
	CHB	206	3.0 ^‡^	2.7 ^‡^	16.0	[20]
	East Asian ^‡^	8064 ^‡^				[21,80] ^‡^
	CEU	198	3.7 ^‡^	3.4 ^‡^	27.8	[20]
	European ^‡^	61,428 ^‡^				[21,80] ^‡^
	ASW	122	6.5 ^‡^	5.8 ^‡^	35.3	[20]
	African ^‡^	8646 ^‡^				[21,80] ^‡^

**Table 6:** Comparison of *CYP2C9* allele frequencies in Indigenous North American populations to global populations from the 1000 Genomes Project [20]. *N* represents the number of alleles. Not shown in the table are CYP2C9*4 (rs56165425) and CYP2C9*6 (rs9332131), as these SNVs were either absent or not tested for in North American Indigenous populations. Global populations are abbreviated as follows: African Ancestry in Southwest US (ASW); Utah residents with Northern and Western European ancestry (CEU); Han Chinese in Beijing, China (CHB); Mexican Ancestry in Los Angeles, California (MXL).

Country	Population	*N*	*CYP2C9* MAF (%)				Refs.
			*2 rs1799853	*3 rs1057910	*5 rs28371686	*29 rs182132442	
Canada	FN	228	3.0	6.0	-	-	[28]
	Inuit	302	0.0	0.0	-	-	[28]
USA	Yup’ik	700	0.3	2.1	0.0	2.1	[33]
	AIAN	718	5.2	3.4	0.0	0.0	[33]
	CSKT	188	5.2	2.7	0.0	0.0	[32]
	AI	246	5.8	2.7	0.4	-	[34]
Mexico	Tepehuano	245	0.0	7.5	-	-	[30]
	Mexicanera	76	0.0	0.0	-	-	[30]
	Huichol	214	0.0	3.3	-	-	[30]
	Seri	38	2.6	0.0	-	-	[30]
	Guarijío	30	0.0	6.7	-	-	[30]
	Mayo	88	5.7	9.1	-	-	[30]
	Cora	162	0.0	3.7	-	-	[30]
	Tarahumara	148	0.0	10.4	-	-	[30]
	Nahua	424	0.7	0.4	-	-	[31]
	Teenek	196	0.5	0.5	-	-	[31]
	Tarahumara	104	0.0	0.0	-	-	[31]
	Purepecha	96	0.0	0.0	-	-	[31]
	Huichol	146	0.0	0.0	-	-	[31]
	Tepehuano	198	1.0	1.5	0.0	-	[29]
	Aggregate of Amerindian tribes	882	0.6	5.1	-	-	[23]
Multiple Countries (1000 Genomes)	MXL	128	10.2	2.3	0.0	0.0	[20]
	CHB	206	0.0	3.9	0.0	0.5	[20]
	CEU	198	15.2	6.6	0.0	0.0	[20]
	ASW	122	4.1	1.6	2.5	0.0	[20]

**Table 7:** Comparison of novel *CYP2C9* allele frequencies identified by full gene resequencing in AN and AI populations, not found in other world populations.

Country	Population	*N*	*CYP2C9* MAF (%)			Refs.
			*M1L* rsNA	*K119T* rsNA	*N218I* rsNA	
USA	Yup’ik	700	6.3	0.0	3.8	[33]
	AIAN	718	1.0	0.0	1.4	[33]
	CSKT	188	0.0	0.57	0.0	[32]

**Table 8:** Comparison of *CYP2C19* allele frequencies in Indigenous North American populations to global populations from the 1000 Genomes Project [20]. For alleles defined by multiple variants, the frequencies reported in Zhou et al. using LDLink software [81] were used. *N* represents the number of alleles. Not shown in the table are CYP2C19*5 (rs56337013) and *CYP2C19*14 (rs55752064), as they were not found in any Amerindian populations and not genotyped for in Indigenous peoples of Canada or AIs.

Country	Population	*N*	*CYP2C19* MAF (%)					Refs.
			*2 rs4244285	*3 rs4986893	*4 rs28399504	*8 rs41291556	*17 rs12248560	
Canada	FN	230	19.1	0.0	-	-	-	[26]
	Inuit	180	12.0	0.0	-	-	-	[35]
USA	Sioux	196	11.2	0.0	-	-	8.7	[36]
	AI	246	11.5	-	1.3	0.0	11.1	[34]
Mexico	Tarahumara	168	31.0	0.0	0.0	-	-	[37]
	Purepecha	202	5.4	0.0	0.0	-	-	[37]
	Tojolabal	136	3.6	0.0	0.0	-	-	[37]
	Tzotzil	176	5.6	0.0	0.0	-	-	[37]
	Tzeltal	40	0.0	0.0	0.0	-	-	[37]
	Aggregate of Amerindian tribes	880	12.0	0.2	0.0	-	2.2	[23]
Multiple Countries (1000 Genomes)	MXL	128	12.5	0.0	0.8	0.0	11.7	[20]
	CHB	206	33.5	4.4	0.5	0.0	2.4	[20]
	CEU	198	13.1	0.0	0.0	1.5	22.2	[20]
	ASW	122	13.9	0.0	0.0	0.8	19.7	[20]

**Table 9:** Comparison of *CYP2D6* allele frequencies Indigenous North American populations to global populations from the 1000 Genomes Project [20]. *N* represents the number of alleles. Not shown is CYP2D6*7 (rs503086), as it was not detected or not tested for in the Indigenous populations studied. Global populations are abbreviated as follows: African Ancestry in Southwest US (ASW); Utah residents with Northern and Western European ancestry (CEU); Han Chinese in Beijing, China (CHB); Mexican Ancestry in Los Angeles, California (MXL). For alleles not captured by 1000 Genomes (noted by ‡), the frequencies were extracted from Exome Aggregation Consortium [80] and for alleles defined by multiple variants, the frequencies reported in Zhou et al. [21] using LDLink software [81] were used.

Country	Population	*N*	*CYP2D6* MAF (%)										Refs.
			*2 rs16947, rs1135840	*3 rs35742686	*4 rs3892097	*5	*6 rs5030655	*8 rs5030865	*10 rs1065852, rs1135840	*17 rs16947, rs28371706	*35 rs769258, rs16947, rs1135840	*41 rs28371725	
Canada	FN	190	-	0.0	3.0	-	-	-	3.0	-	-	-	[39]
	Inuit	180	-	0.0	6.7–8.3	-	-	-	2.2	-	-	-	[38]
USA	AI	246	25.2	0.0	14.6	2.8	0.8	-	2.0	0.0	0.4	6.9	[34]
	CSKT	374	23.4	0.3	20.9	1.3	0.0	-	1.3	0.0	1.1	11.2	[32]
Mexico	Tepehuano	198	20.0	0.0	0.6	0.5	0.0	-	0.0	-	0.0	1.0	[41,42]
	Tarahumara	176	-	0.0	7.3	-	0.0	0.0	-	-	-	-	[37]
	Purepecha	170	-	0.0.	2.9	-	0.0	0.0	-	-	-	-	[37]
	Tojolabal	86	-	0.0	1.2	-	0.0	0.0	-	-	-	-	[37]
	Tzotzil	112	-	0.0	2.7	-	0.0	0.0	-	-	-	-	[37]
	Tzeltal	38	-	0.0	5.3	-	0.0	0.0	-	-	-	-	[37]
	Tarahumara	148	21.0	0.0	11.5	3.4	0.0	-	0.7	0.0	0.0	4.1	[43]
	Tepehuano	258	20.0	0.0	0.3	0.4	0.0	-	0.0	0.0	0.0	0.4	[43]
	Huichol	214	21.0	0.0	7.0	0.0	0.0	-	0.0	0.0	0.0	0.0	[43]
	Mexicanera	78	22.0	0.0	0.0	1.3	0.0	-	0.0	0.0	0.0	0.0	[43]
	Cora	162	28.0	0.0	1.0	1.2	0.0	-	0.0	0.0	0.0	1.0	[43]
	Seri	38	5.0	0.0	21.0	0.0	0.0	-	0.0	0.0	0.0	0.0	[43]
	Guarijío	30	23.0	0.0	3.0	0.0	0.0	-	0.0	0.0	0.0	0.0	[43]
	Mayo	88	10.0	0.0	8.0	0.0	3.0	-	0.0	0.0	0.0	3.0	[43]
	Mayan Lacandon	308	20.8	0.0	10.4	0.0	0.0	-	0.6	0.0	0.3	1.3	[44]
	Tzotzil and Tzeltal	220	17.3	0.5	5.5	3.6	0.0	-	0.5	0.5	0.0	0.0	[45]
	Aggregate of Amerindian tribes	758	18.9	-	4.5	1.0	0.1	-	0.1	-	-	1.1	[23]
Multiple Countries (1000 Genomes)	MXL	128	-	0.0	12.5	-	0.0	0.0	-	-	-	1.6	[20]
	Latino ^‡^	9768 ^‡^											[80] ^‡^
	CHB	206	14.0 ^‡^	0.0	0.5	6.5 ^‡^	0.0	0.5	58.7 ^‡^	0.0 ^‡^	-	3.4	[20]
	East Asian ^‡^	7968 ^‡^											[21,80] ^‡^
	CEU	198	34.3 ^‡^	2.0	22.7	3.0 ^‡^	2.0	0.0	0.2 ^‡^	<0.1 ^‡^	-	12.1	[20]
	European ^‡^	56,352 ^‡^											[21,80] ^‡^
	ASW	122	26.7 ^‡^	1.6	12.3	4.0 ^‡^	0.8	0.0	3.2 ^‡^	19.7 ^‡^	-	1.6	[20]
	African ^‡^	7304 ^‡^											[21,80] ^‡^

**Table 10:** Comparison of *CYP2E1* allele frequencies Indigenous North American populations to global populations from the 1000 Genomes Project [20]. *N* represents the number of alleles. Global populations are abbreviated as follows: African Ancestry in Southwest US (ASW); Utah residents with Northern and Western European ancestry (CEU); Han Chinese in Beijing, China (CHB); Mexican Ancestry in Los Angeles, California (MXL).

Country	Population	*N*	*CYP2E1* MAF (%)		Refs.
			*1D	*5B rs2031920	
Canada	FN	228	9.3	-	[46]
Mexico	Huichol	198	-	51.5	[47]
Multiple Countries (1000 Genomes)	MXL	128	-	15.6	[20]
	CHB	206	-	23.8	[20]
	CEU	198	-	6.1	[20]
	ASW	122	-	1.6	[20]

**Table 11:** Comparison of *CYP3A4* allele frequencies Indigenous North American populations to global populations from the 1000 Genomes Project [20]. *N* represents the number of alleles. Not shown are CYP3A4*2 (rs55785340), CYP3A4*4 (rs55951658) and CYP3A4*8 (72552799), as these variants were either not detected or not tested for in the Indigenous populations studied. Global populations are abbreviated as follows: African Ancestry in Southwest US (ASW); Utah residents with Northern and Western European ancestry (CEU); Han Chinese in Beijing, China (CHB); Mexican Ancestry in Los Angeles, California (MXL).

Country	Population	*N*	*CYP3A4* MAF (%)				Refs.
			*1B rs2740574	*1G rs2242480	*5 rs55901263	*22 rs35599367	
USA	CSKT	188	2.2	26.8	0.0	2.4	[32]
Mexico	Tepehuano	200	8.0	-	0.0	-	[48]
	Aggregate of Amerindian tribes	420	4.8	-	-	-	[23]
Multiple Countries (1000 Genomes)	MXL	128	7.0	39.1	0.0	0.8	[20]
	CHB	206	0.0	24.8	0.5	0.0	[20]
	CEU	198	1.5	5.6	0.0	4.6	[20]
	ASW	122	67.2	74.6	0.0	0.0	[20]

**Table 12:** Comparison of *CYP3A5* allele frequencies Indigenous North American populations to global populations from the 1000 Genomes Project [20]. *N* represents the number of alleles. Global populations are abbreviated as follows: African Ancestry in Southwest US (ASW); Utah residents with Northern and Western European ancestry (CEU); Han Chinese in Beijing, China (CHB); Mexican Ancestry in Los Angeles, California (MXL).

Country	Population	*N*	*CYP3A5* MAF (%)			Refs.
			*3 rs776746	*6 rs10264272	*7 rs41303343	
USA	CSKT	188	92.5	0.0	0.0	[32]
Multiple Countries (1000 Genomes)	MXL	128	76.6	2.3	0.0	[20]
	CHB	206	68.9	0.0	0.0	[20]
	CEU	198	96.0	0.0	0.0	[20]
	ASW	122	31.2	4.9	12.3	[20]

**Table 13:** Comparison of *CYP4F2* allele frequencies Indigenous North American populations to global populations from the 1000 Genomes Project [20]. *N* represents the number of alleles. Global populations are abbreviated as follows: African Ancestry in Southwest US (ASW); Utah residents with Northern and Western European ancestry (CEU); Han Chinese in Beijing, China (CHB); Mexican Ancestry in Los Angeles, California (MXL).

Country	Population	*N*	*CYP4F2* MAF (%)					Refs.
			*2 rs3093105	*3 rs2108622	*G185V* rs3093153	*L519M* rs3093200	*spliceCG* rsNA	
USA	Yup’ik	700	3.7	50.9	0.3	0.0	0.7	[33]
	AIAN	718	11.0	31.5	2.2	2.7	1.4	[33]
Multiple Countries (1000 Genomes)	MXL	128	14.1	25.0	1.6	0.8	-	[20]
	CHB	206	8.3	21.8	0.0	0.0	-	[20]
	CEU	198	14.1	24.8	7.0	8.6	-	[20]
	ASW	122	25.4	9.0	0.8	13.9	-	[20]
